# Exercise to Improve Mobility in Healthy Aging

**DOI:** 10.1007/s40279-015-0405-9

**Published:** 2015-09-23

**Authors:** Urs Granacher, Tibor Hortobágyi

**Affiliations:** Division of Training and Movement Sciences, University of Potsdam, Potsdam, Germany; Center for Human Movement Sciences, University of Groningen, University of Groningen Medical Center, Groningen, The Netherlands; Faculty of Health and Life Sciences, Northumbria University, Newcastle upon Tyne, UK

Biological aging inevitably affects every individual, no matter what their color of skin, country of origin, level of education, and socio-economic status is. Intuitively, we associate aging with old age. However, aging encompasses more than senescence. In fact, the process begins at conception and continues throughout the lifespan, with marked physiological and performance changes occurring for some reason after age 60 years. Aging is a process or group of processes occurring in living organisms that with the passage of time leads to a loss of adaptability, functional impairment, and eventually death [1]. Healthy aging is a desirable path that is worth pursuing for every individual. Factors that determine healthy or successful aging are avoiding disease (e.g., obesity), engagement with life (e.g., social activities), maintaining high cognitive and physical function through, for instance, proper diet, and sufficient physical activity [1]. The American College of Sports Medicine and the American Heart Association have provided recommendations on the types and amounts of physical activity needed to improve and maintain health in older adults [2]. With this purpose in mind, seniors are advised to perform physical activity at a moderate intensity that results in aerobic metabolism for a minimum of 30 min on 5 days each week or vigorous-intensity aerobic activity for a minimum of 20 min on 3 days each week. To maintain the flexibility necessary for regular physical activity and daily life, older adults should perform activities that maintain or increase flexibility on at least 2 days each week for at least 10 min each day. To reduce the risk of injury due to falls, community-dwelling older adults at substantial risk of falls should perform exercises that maintain or improve balance. In addition, at least twice each week older adults should perform muscle-strengthening activities using the major muscles of the body to maintain or increase muscular strength and endurance [2].

The German Research Foundation provided support for a series of workshops that took place in Potsdam, Freiburg, and Kassel between October 2013 and September 2014 (MU 3327/2-1). International experts discussed current knowledge of how age affects motor performance throughout the lifespan and how exercise interventions could improve motor performance in older adults, delay the onset and progression of mobility disability, and promote healthy aging. To complement already existing physical activity guidelines, this themed issue of *Sports Medicine* contains systematic literature reviews and meta-analyses that provide evidence-based overviews of current knowledge concerning the efficacy of exercise interventions with a focus on seniors. Four of seven papers focus on the intervention effects of strength training using unstable surfaces, resistance training, power training, coordination training, and balance training on measures of balance, strength, power, and mobility in cohorts across the lifespan with a focus on older adults (Behm et al., Borde et al., Hortobágyi et al., Lesinski et al). Two papers describe the dose-response relationships following resistance and balance training in older adults (Borde et al., Lesinski et al.). This is new and important evidence-based information for practitioners and therapists seeking to design and implement effective exercise programs to counteract dynapenia, sarcopenia, and mobility disability in seniors. Further, two papers analyze how task difficulty affects responses to balance perturbations in older adults (Bohm et al.) and cognitive-motor dual tasking across the lifespan (Ruffieux et al.), with a perspective on intervention design. Finally, Muehlbauer et al. examine the relationship between measures of balance and leg strength/power across the lifespan in an effort to provide information that facilitates better-informed exercise prescription.

The consistent message from this wealth of up-to-date information is that spontaneous physical activity and systematic exercise delivered in a variety of forms can increase motor performance independent of age, slow the progression of mobility disability, and most likely improve health in old age. As part of their efforts to promote healthy aging, practitioners, healthcare professionals, and policy makers can use this new knowledge to recommend and urge seniors to become and remain physically active.
